# Virome characterization of diarrheic red-crowned crane (*G. japonensis*)

**DOI:** 10.1186/s42523-024-00299-3

**Published:** 2024-02-28

**Authors:** Ning Cui, Xiao Yang, Hong Sui, Liugang Tan, Weihua Wang, Shuai Su, Lihong Qi, Qinghua Huang, Nataliia Hrabchenko, Chuantian Xu

**Affiliations:** 1 Shandong Key Laboratory of Animal Disease Control and Breeding, Institute of Animal Science and Veterinary Medicine, Shandong Academy of Agricultural Sciences, Jinan, China; 2 Key Laboratory of Livestock and Poultry Multi-omics of MARA, Jinan, China; 3 Shandong Provincial Key Laboratory of Animal Biotechnology and Disease Control and Prevention, College of Veterinary Medicine, Shandong Agricultural University, Tai’an, China; 4 Dongying Animal Husbandry and Veterinary Station, Dongying, China; 5 National Nature Reserve Management Committee of Shandong Yellow River Triangle, Dongying, China

**Keywords:** *Grus japonensis*, Diarrhea, Virome, Megrivirus A, Genomovirus, Parvovirus

## Abstract

**Background:**

The red-crowned crane is one of the vulnerable bird species. Although the captive population has markedly increased over the last decade, infectious diseases can lead to the death of young red-crowned cranes while few virological studies have been conducted.

**Methods:**

Using a viral metagenomics approach, we analyzed the virome of tissues of the dead captive red-crowned crane with diarrhea symptoms in Dongying Biosphere Reserve, Shandong Province, China and feces of individual birds breeding at the corresponding captive breeding center, which were pooled separately.

**Results:**

There is much more DNA and RNA viruses in the feces than that of the tissues. RNA virus belonging to the families *Picornaviridae*, and DNA viruses belonging to the families *Parvoviridae*, associated with enteric diseases were detected in the tissues and feces. Genomes of the picornavirus, genomovirus, and parvovirus identified in the study were fully characterized, which further suggested that infectious viruses of these families were possibly presented in the diseased red-crowned crane.

**Conclusion:**

RNA virus belonging to the families *Picornaviridae*, and DNA viruses belonging to the families *Genomoviridae* and *Parvoviridae* were possibly the causative agent for diarrhea of red-crowned crane. This study has expanded our understanding of the virome of red-crowned crane and provides a baseline for elucidating the etiology for diarrhea of the birds.

## Introduction

Red-crowned crane (*Grus japonensis*) is a species of large, wading, omnivorous bird in the family *Gruidae* (*cranes*). The red-crowned crane is one of the National Class I Key Wildlife Protection of China’s National List of Key Wildlife Protection and listed as a vulnerable bird species by the International Union for Conservation of Nature (IUCN 2021). The current population in the wild globally is estimated to be 2800–3430 individuals [1]. Several efforts, including the creation of biosphere reserves and captive breeding programs, have been made to maintain number of communities by reintroducing captive-bred cranes into the wild. Although the captive population has markedly increased over the last decade [2], infectious diseases can lead to the death of young red-crowned cranes while few virological studies have been conducted [3].

Recently, our understanding of the virosphere has been revolutionized bymetagenomics [4]. Metagenomic next-generation sequencing (mNGS), independent of culture, has massively fostered the rate of virus discovery by identifying divergent viruses that could not be detected using traditional approaches. In addition, these technical advances also enable the assembly of the viral genome quickly. Importantly, the diversity and abundance of viruses in individuals or environmental samples can be achieved using NGS-based approaches followed by intensive bioinformatics analyses. Using a viral metagenomics approach, virome of feces from wild and captive red-crowned cranes has been investigated and identified a large numbers of vertebrate, plant, insect, and aquatic animal viruses [5].

The current study was designed to detect and characterize the virome presented in captive breeding birds in Dongying Biosphere Reserve in Shandong Province, which is located at the Chinese eastern migration area for bird migration. Specifically, we wanted to explore the possible viral etiology for the diarrheal death of the captive red-crowned cranes breeding in the biosphere reserve.

## Materials and methods

### Sample collection and preparation

**Fig. 1:**
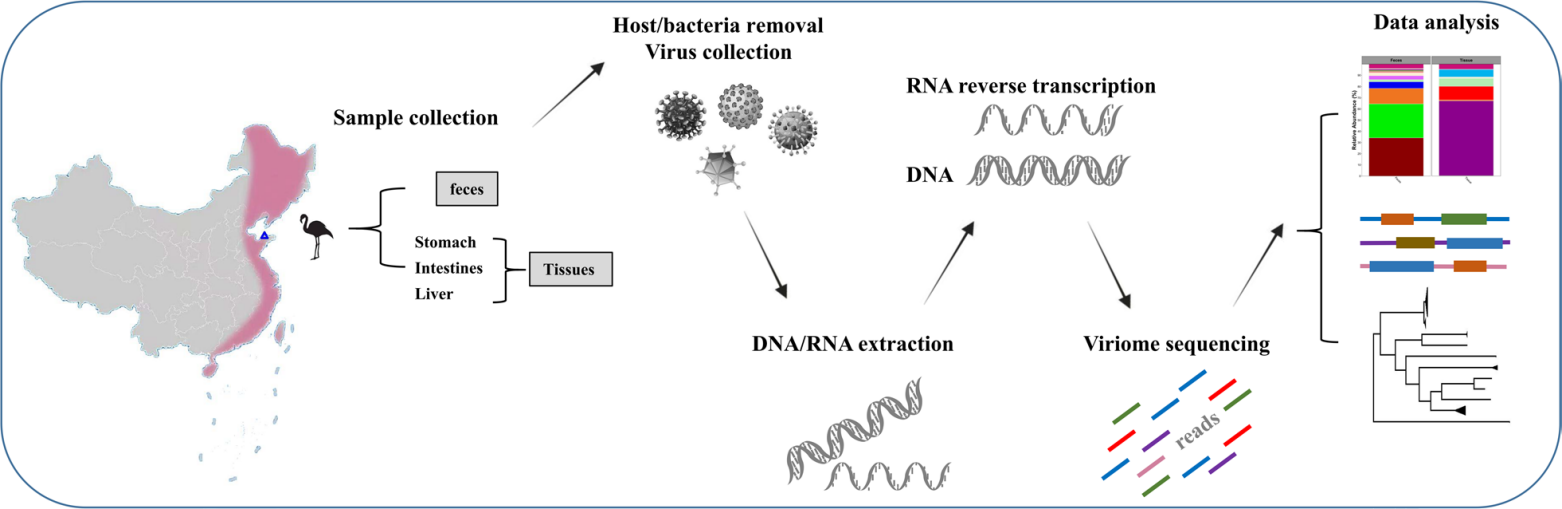
Graphical scheme and flow chart of experiment design for virus discovery. Tissues and feces samples were collected from Dongying Biosphere Reserve, Shandong Province, China in spring, 2022. The samples were pooled separately and subjected for the following host/bacteria removal and virus collection, DNA/RNA extraction, viriome sequencing and final bioinformatics analysis

In the spring of 2022, some 3-month-old red-crowned cranes, raised in Dongying Biosphere Reserve, Shandong Province, China, showed symptoms of acute watery diarrhea, and one died within a day without any bleeding and tissue lesions in autopsy examination. Tissue sample containing stomach, the upper part of the intestines and liver of the dead red-crowned crane was collected immediately and stored on dry ice prior to shipping to the laboratory (Fig. 1). Feces sample was a pool of thirty fresh feces from individual clinically healthy birds of different ages breeding at the captive breeding center, and the feces of wild birds were not excluded. About 2 g of each tissues were collected respectively, cut into small pieces and pooled together, inverted mixed gently and then incubated in 1 ml digestion buffer (1 ml DPBS, 1 mg/ml collagenase I, 12 U DNase I, pH 7.5 ) at 37 °C for 1 h. Then, the digested samples were centrifugated at 5000 × g for 10 min to remove debris, cells and bacteria. After that, the supernatants were passed through 0.22 μm filters (Sartorius). The filtrates were treated with 200U Benzonase (Millipore), Turbo DNase I (ThermoFisher Scientific) and 0.1 mg/ ml RNase A (Sangon Biotech) followed by heat inactivation of DNases at 65 °C for 10 min. Fecal sample (~ 200 mg) was suspended in 1.2 mL saline-magnesium buffer, and vortexed for 10 min followed by centrifugation at 5000 × g for 20 min. The clarified suspensions were filtered through 0.22 μm filters (Sartorius). The filtrates were treated with 200U Benzonase (Millipore) and 0.1 mg/ml RNase A (Sangon Biotech) followed by heat inactivation of DNases at 65 °C for 10 min.

### Virome sequencing

Virome sequencing was performed in Chengdu Life Baseline Technology Co., Ltd.Viral RNA and DNA extraction was performed with QIAgen MinElute Virus Spin Kit according to the manufacture’s recommendation. The DNA concentration was quantified using Qubit with a Equalbit 1× dsDNA HS Assay Kit (Vazyme Biotech Co., Ltd), and the RNA concentration was determined using Qubit with a Equalbit RNA HS Assay Kit (Vazyme Biotech Co., Ltd). The virome library was constructed using a sequence independent Amplification (SIA) method [6]. Briefly, the SIA was facilitated on the total nucleic acid using 200U superscript III reverse transcriptase with a random primer (5′-GACCATCTAGCGACCTCCACNNNNNNNN-3′) followed by the second strand synthesis with 2.5 U 3′–5′ exo-Klenow DNA polymerase (New England Biolabs). Then, the double strand product was PCR amplified with a specific primer (5′-GACCATCTAGCGACCTCCAC-3′). The purified amplification product was fragmented, ends repaired, dA-tailed, adaptor ligated and amplified with VAHTS® Universal Plus DNA Library Prep Kit for Illumina (Vazyme Biotech Co., Ltd). The library was quality-checked by an Agilent4200 Bioanalyzer and sequencing was performed on the Illumina Nova Seq 6000platform with 2 × 150 bp pair-end reads.

### Data analyses

Fastp was used to remove the low-quality reads or reads containing a high proportion of N and cut the adapters to obtain clean reads after the sequencing [7]. The rRNA sequences and host contamination in fecal sample was removed by Bowtie2 [8] with a collection of genomic sequences including *Anser fabalis*, *Fulica atra*, *Ciconia boyciana*, *Ardea cinerea*, *Aix galericulata*, *Egretta garzetta*, *Grus japonensis*, *Anser cygnoides*, *Anas platyrhynchos*. For tissue sample, the genome of *Grus japonensis* was used. MEGAHIT [9] was used to the de novo assembly to obtain the contigs (-min-contig-len 500). Open reading frames (ORFs) was then predicted by Prodigal [10]. Viral contigs were identified by database comparison (NR database and RdRp database) and virus identification software (Virsorter2 [11], Deep virfinder [12], and VIBRANT [13]) simultaneously, and the union set were used for subsequent analysis. BBSketch and hmmsearch was used to remove false positive sequences of non-viral species, such as genome sequences of bacteria, fungi, and protozoa. High-Similarity (HS) annotation viral contigs with the nucleic acid similarity and protein similarity more than 85% were considered to be a high confidence virus sequence, which will be used for further virus sequence assembly.

### Phylogenetic analyses for identified viruses

To generate phylogenetic trees, the assembled genome sequence of each virus was subjected to a BLASTN search against NCBI’s GenBank (http://blast.ncbi.nlm.nih.gov/Blast.cgi). We retrieved the top homologous viral genome sequences and the representative strains of different species from corresponding family for individual virus. Nucleotide sequences were aligned by ClustalW program [14]. Phylogenetic trees were constructed by MEGA6 program [15] using the neighbor-joining method with a bootstrap of 1000 replicates as previously reported [16].

## Results

### Overview of metagenomic data and virome composition

In our study, 66,814,680 clean reads were obtained in feces sample and 67,924,496 clean reads were obtained in tissues sample, among them 135,636 (0.20%) and 79,818 (0.12%) were viral reads. A total of 10,899 and 685 viral contings were identified in feces and tissues sample respectively (Table 1), indicating there is much more viruses in the feces than that of the tissues. For feces sample, 22 viral contings were defined as HS annotation RNA viruses and 28 viral contings were defined as HS annotation DNA viruses. For tissues sample, only 5 viral contings were defined as HS annotation RNA viruses and 1 viral contings were defined as HS annotation DNA viruses. In addition, 1118 and 104 novel viruses were predicated in feces and tissues sample respectively.

**Table 1:** Summary of the metagenomic data

Sample	Clean reads	Viruses reads (percentage)	Viral contigs	GC content (%)	ORFs	RNA viruses	HS annotation RNA viruses	DNA viruses	HS annotation DNA viruses	PHG viruses	HS annotation PHG viruses	Novel viruses
Feces	66,814,680	135,636 (0.20%)	10,899	48.41	31,947	120	22	583	28	9078	808	1118
Tissues	67,924,496	79,818 (0.12%)	695	48.84	1712	17	5	9	1	285	104	104

*ORFs* Open reading frames, *HS* High-similarity, *PHG* Phage

**Fig. 2:**
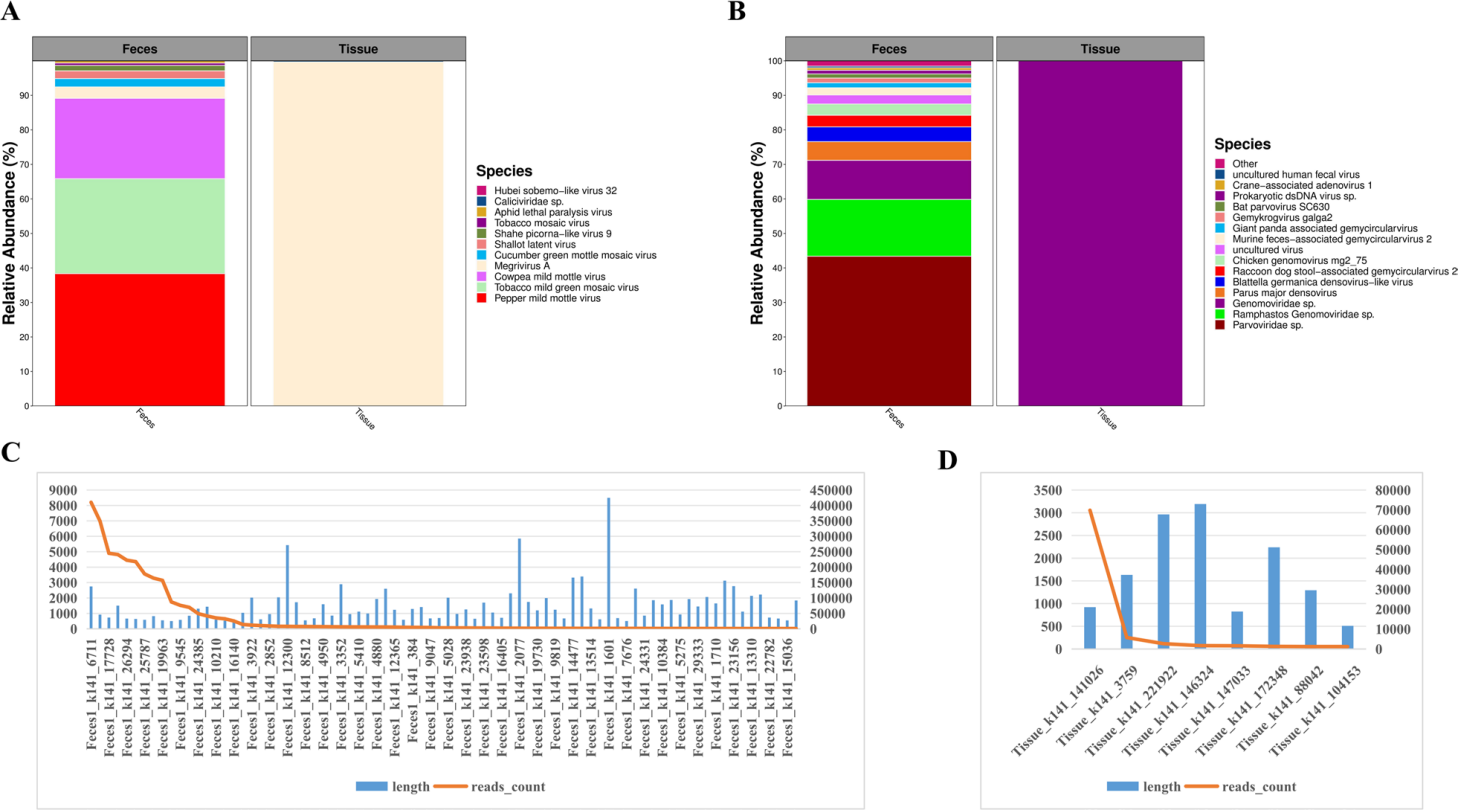
Overview of the virus composition. Relative abundance of RNA viruses (**A**) and DNA viruses (**B**), novel viral reads (**C**, **D**) in the feces and tissues respectively based on the viral contig numbers

RNA viruses (Fig. 2A) including Pepper mild mottle virus, Tobacco mild green mosaic virus, Cowpea mild mottle virus, Megrivirus A, Cucumber green mottle mosaic virus, Shallot latent virus, Shahe picorna-like virus 9, Tobacco mosaic virus, Aphid lethal paralysis virus, Hubei sobemo-like virus 32, *Caliciviridae sp*., and DNA viruses (Fig. 2B) including *Parvoviridae sp*., *Ramphastos Genomoviridae sp*. *Genomoviridae sp*., Parus major densovirus, Blattella germanica densovirus-like virus, Raccoon dog stool-associated gemycircularvirus 2, Chicken genomovirus mg2_75, uncultured virus, Murine feces-associated gemycircularvirus 2, Giant panda associated gemycircularvirus, Gemykrogvirus galga2, Bat parvovirus SC630, *Prokaryotic dsDNA virus sp*., Crane-associated adenovirus 1, uncultured human fecal virus, Finch associated genomovirus 7, uncultured marine virus, Lepidopteran ambidensovirus 1, uncultured T7-like podovirus, Gemycircularvirus mocha1, *CRESS virus sp*. were the found in feces. However, only Megrivirus A and *Genomoviridae sp*. were found in tissues, both of which were also detected in feces. Consistent with the overview of metagenomic data, the feces contains more virus species than that of the tissues. The same is true of the novel viruses that the feces sample possessed 80 novel viruses (Fig. 2C, three of the nucleotide sequence length greater than 5000 nt) with reads count more than 1000, ten times as many novel viruses as the tissues sample (Fig. 2D).

### Genome architectures and evolution of picornavirus

**Fig. 3:**
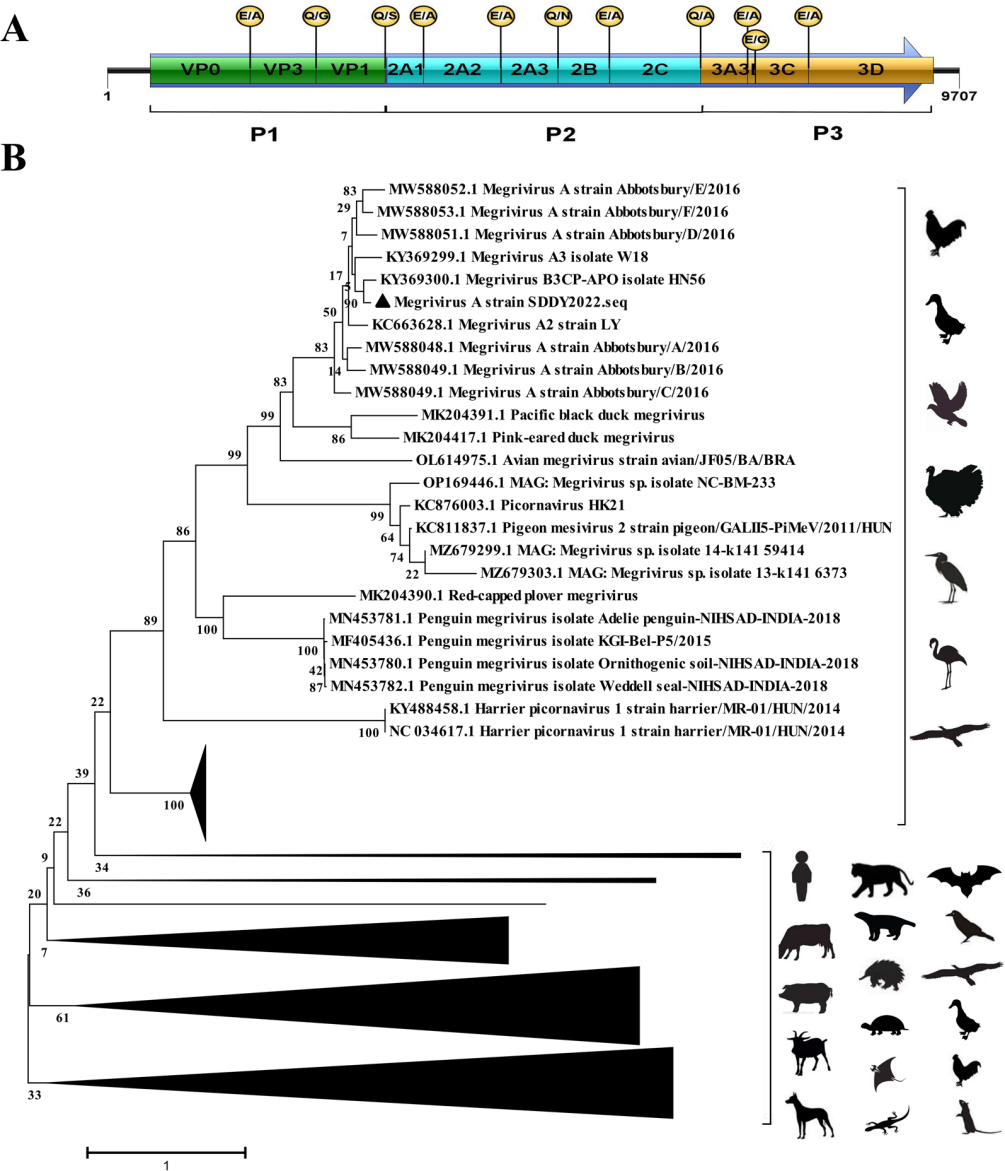
Genomic organization and phylogenetic analysis of the Megrivirus A of ***Picornaviridae***. **A** Genomic organization of the Megrivirus A strain indicating open reading frames (ORFs). The predicated polypeptides were shown below the gene box and the the predicted cleavage sites are shown above the gene box. **B** Phylogenetic relationships of Megrivirus A with other viruses in the *Picornaviridae* family based on the complete genomes. Phylogenetic tree was constructed using the MEGA6 program using the Neighbouring method with a bootstrap of 1000 replicates. Virus identified in this study is denoted with a black filled triangle

Picornaviruses were found in both samples. The family *Picornaviridae* currently consists of 158 species grouped into 68 genera (as of July 2022). In this study, a library of the tissues containing 3977 sequence reads related to *Picornaviridae* sp. could be assembled into a nearly complete genome of picornavirus (9707 nt) with 89.13% identity and 99% coverage with Megrivirus HN56 (GenBank no. KY369300). The picornavirus strain was named SDDY2022 and included a 491-bp 5′ UTR, a 8919-bp polyprotein ORF encoding putative polyprotein of 2972 aa and a 297-bp partial 3′ UTR (Fig. 3A). Phylogenetic analysis based on the complete genomes of representative strains from the family *Picornaviridae* showed that the SDDY2022 strain was clustered with two goose Megriviruses (W18 and HN56) causing enteritis, duck megrivirus strain LY, and a cluster of Megrivirus A strains identified from Cygnus olor in United Kingdom, all of them were belonging to the Megrivirus A branch that infecting birds (Fig. 3B). Based on sequence alignment with the closet HN56 strain, P1, P2, and P3 polypeptides that showing 97.1%, 95.7%, and 98.8% homology were identified in the polyprotein. The P1 polypeptide (892aa) was assumed to be cleaved at VP0/VP3 (^378^E/A), VP3/VP1 (^629^Q/G) to produce three capsid proteins. The P2 polypeptide (1196aa) and P3 polypeptide (884aa) was assumed to be cleaved at 2A1/2A2 (^1037^E/A), 2A2/2A3 (^1331^E/A), 2A3/2B (^1548^Q/A), 2B/2C (^1743^E/A), 2 C/3A (^2088^Q/A), 3A/3B (^2267^E/A), 3B/3C (^2296^E/G), and 3 C/3D (^2498^E/A) to produce nine nonstructural proteins. The SDDY2022 and HN56 strains possessed the same cleavage sites as above, except that the cleavage site between P1 polypeptide and P2 polypeptide was ^892^Q/S, while it was ^892^Q/N in HN56 strain. Together these data suggest that the tissues sample contains Megrivirus A with typical picornavirus structure. In addition, three partial sequences in tissues and feces were also identified respectively.

### Genome architectures and evolution of genomovirus

**Fig. 4:**
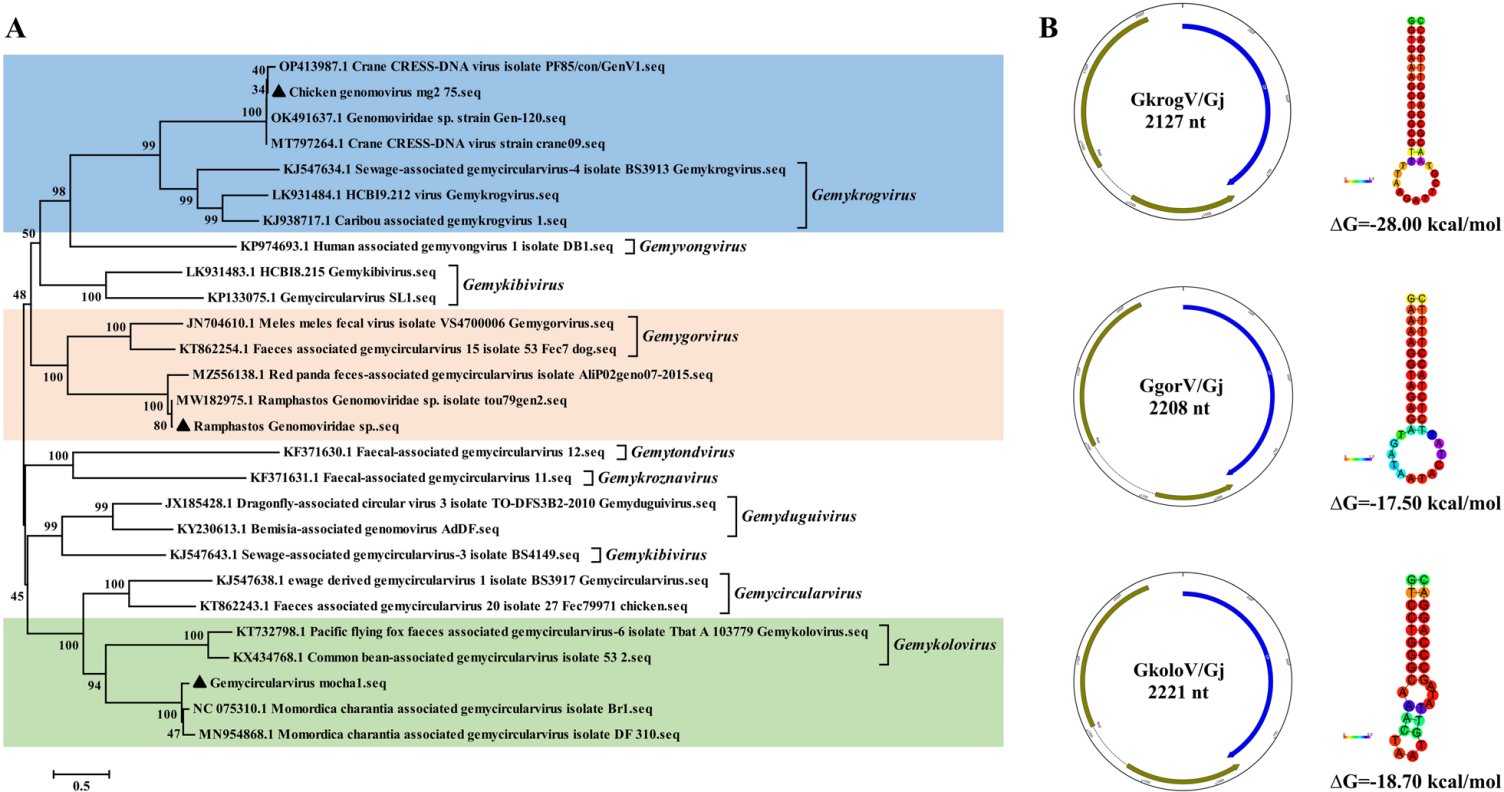
Phylogenetic analysis and genomic features of the identified three genomoviruses. **A** Phylogenetic relationships of the identified genomoviruses with other viruses of ten genera of the family *Genomoviridae* based on the complete genomes. Phylogenetic tree was constructed using the MEGA6 program using the Neighbouring method with a bootstrap of 1000 replicates. Viruses identified in this study are denoted with a black filled triangle. **B** Genomic organization indicating ORFs and stem-loop structures of the identified genomoviruses

A large number of genomovirus sequence reads were detected in the feces samples. The family *Genomoviridae* currently consists of 237 species grouped into 10 genera (as of July 2022). Three complete genome sequences of genomovirus, named GkrogV/Gj, GgorV/Gj, and GkoloV/Gj, were assembled from feces. GkrogV/Gj was 98.40% identity to Genomoviridae sp. strain Gen-120 (GenBank no. OK491637) with 100% coverage, GgorV/Gj was 99.91% identity to Genomoviridae sp. isolate 190Gen-2 (GenBank no. OM892309) with 100% coverage, and GkoloV/Gj was 99.91% identity to Momordica charantia associated gemycircularvirus isolate Br1 (GenBank no. NC_075310) with 93% coverage. Phylogenetic analysis based on the complete genomes of the representative strains from the family *Genomoviridae* showed that they were closely related to the genus of *Gemykrogvirus*, *Gemygorvirus*, and *Gemykolovirus* respectively (Fig. 4A). The complete genome sizes were 2127, 2208, and 2221nt in length, all of which encoded two major proteins: capsid protein (Cp) and replication-associated protein (Rep), including a putative intron (Fig. 4B). Non-canonical stem-loop structures were identified in the genome of genomoviruses. In addition, seven partial sequences associated finch, Raccoon dog, murine, Giant panda were also identified in feces, while only a partial sequence belonging to *Genomoviridae sp*. was identified in tissues.

### Genome architectures and evolution of parvovirus

**Fig. 5:**
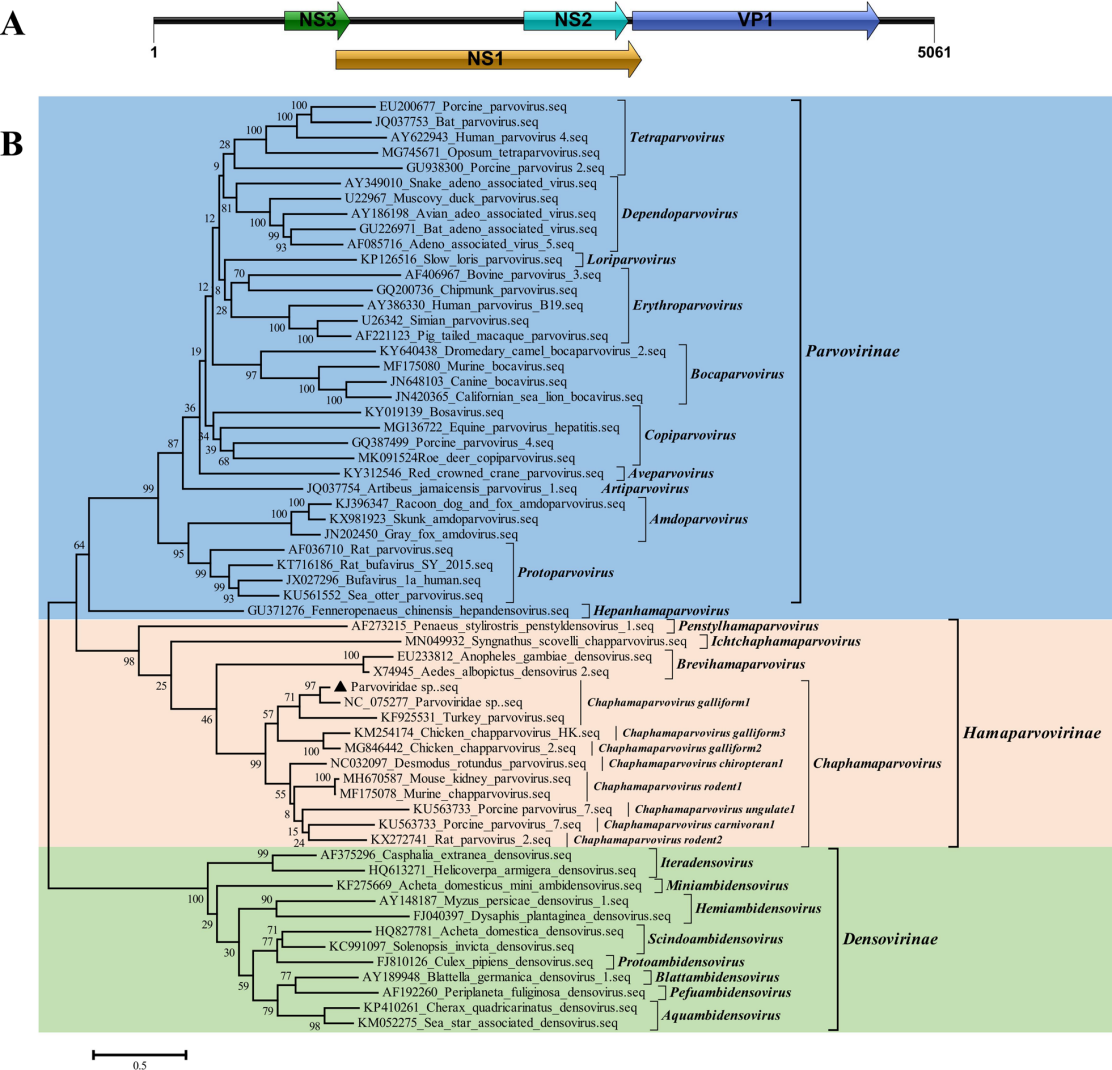
Genomic organization and phylogenetic analysis of the parvovirus. **A** Genomic organization of the identified parvovirus indicating ORFs. **B** Phylogenetic relationships of parvovirus with representative strains of the family *Parvoviridae* based on the complete sequence of NS1 gene. Phylogenetic tree was constructed using the MEGA6 program using the Neighbouring method with a bootstrap of 1000 replicates. Virus identified in this study is denoted with a black filled triangle

In addition to genomovirus, a large number of parvovirus sequence reads were also detected in the feces samples.Viruses within the family *Parvoviridae* are currently grouped into three phylogenetically defined subfamilies and an undefined genus (*Metalloincertoparvovirus*): *Parvovirinae* (11 genera), which includes thus far only viruses infecting vertebrates; *Densovirinae* (11 genera), which comprises viruses infecting invertebrates; and *Hamaparvovirinae*, a recently established taxon that contains viruses identified in both vertebrate (2 genera) and invertebrate (3 genera) host(as of July2022). A complete genome sequence of parvovirus, named ChapV/g1/Gj, was acquired from 22,984 reads. The assembled genome of the parvovirus was 5601nt in length, which was 83.96% identity to Parvoviridae sp. isolate yc-10 (GenBank no. NC_075277) with 78% coverage, and contain four putative ORFs encoding non-structural proteins NS1, NS2, NS3 (P15 accessory protein) at the left end, and a viral capsid protein VP1 at the right end (Fig. 5A). Phylogenetic analysis based on the complete sequence of NS1 (100% coverage) from representative strains of the family *Parvoviridae* showed that the identified parvovirus in the present study, as well as *Parvoviridae sp.* isolate yc-10, belongs to the species *Chaphamaparvovirus galliform1* of genus *Chaphamaparvovirus* of subfamily *Hamaparvovirinae* (Fig. 5B). In addition, six partial sequences belonging to subfamily *Densovirinae* associated wild and zoo birds including Blattella germanica, Parus major, Junonia coenia, and one partial sequence belonging to subfamily *Parvovirinae* associated bat were also identified in feces.

## Discussion

Enteric disease is an ongoing problem worldwide. Gastrointestinal symptoms are caused by several factors, such as infecting viruses. Wild birds may harbor a large number of pathogens that may cause diseases in animals or humans [17, 18]. Many enteric viruses have been identified in birds with the application of mNGS method independent of traditional culture [19]. Multiple co-infections promote and facilitate the recombination and evolution of viruses and eventually could contribute to the severity of the diarrhea. Herein, we employed mNGS method to characterize the virome of the tissue of a young dead red-crowned crane with diarrhea symptoms and a pool of fresh feces from individual birds breeding at the corresponding captive breeding center. Consistent with previous studies, the feces of red-crowned crane contains abundant both DNA and RNA viruses [5]. Collectively, two major families of DNA virus groups from *Genomoviridae* and *Parvoviridae* were identified in the feces. Except for Megrivirus A of family *Picornaviridae* infecting animals, numerous RNA viruses believed to infect insects, plants, and crustaceans were dominated in the feces, which have also been found in the aquatic environment, feces of human and various animals, and probably derived from the diet of red-crowned cranes [5]. *Parvoviridae* virus and Megrivirus A were the dominate DNA and RNA virus found in tissues of the diseased red-crowned crane, both of which were also detected in feces. Therefore, virus species from families *Picornaviridae*, *Genomoviridae* and *Parvoviridae* maybe the causative agents trigger diarrhea symptoms of the red-crowned crane. Moreover, a nearly complete genome of picornavirus, three complete genome sequences of genomovirus, and a complete genome sequence of parvovirus were assembled. It further suggested that infectious viruses of families *Picornaviridae*, *Genomoviridae* and *Parvoviridae* were highly possibly presented in the diseased red-crowned crane.

Members of picornaviruses contains diverse non-enveloped, positive ssRNA viruses with a genome of 7–9 kb in length,which may cause various diseases in different vertebrate hosts, including enteric diseases [20, 21]. Of particular note is the discovery of picornaviruses both in the tissues and feces samples in this study, and a nearly complete genome of picornavirus was identified in the tissues. The identified picornavirus was clustered with two goose Megriviruses (W18 and HN56) causing enteritis [22], both of which were belonging to the Megrivirus A branch that infecting birds. W18 and HN56 was identified from goose flocks with approximately 20% death in 15- to 30-day-old geese, and suspected as a potential recombinant virus, with a distinct P1 region possibly originating from an unknown picornavirus [22]. P1, P2, and P3 polypeptides of the SDDY2022 strain in the study were highly homology with HN56, and the two viruses shared nearly all the same cleavage sites except one in the P2 polypeptide. The results demonstrated that the SDDY2022 strain may be came from a common ancestor virus with HN56. Our results and previous report on HN56 together verified that the Megrivirus A could be an important enteric virus, which may be the major causative agent trigger diarrhea symptoms in the red-crowned crane in our study. In addition, these viruses also have a close relationship with duck megrivirus strain LY that was highly prevalent in duck populations [23], and a cluster of Megrivirus A strains identified from Cygnus olor in United Kingdom. These findings suggest that Megrivirus A infections were geographically widespread around the world, and wild waterbirds may play an important role in the spread and recombination of picornaviruses.

The *Genomoviridae* family, which includes viruses with small, circular ssDNA genomes(~ 2-2.4 kb), encodes two proteins—Cp and Rep—and an intergenic region [24]. In the present study, the feces sample is rich in *Genomoviridae sp*., while these viruses were restricted in the reads but dominant in the tissues sample. A large number of uncultivated *Genomoviridae* viruses have been found in association with a great variety of environmental, plant, and animal samples with the advent of metagenomics approaches [25]. However, no direct implication with a disease has been demonstrated so far. Previously, complete genome sequences of gemycircularvirus have been assembled from both wild and breeding red-crowned cranes [5]. Herein, a total of three complete genome sequences closely related to genus *Gemykrogvirus*, *Gemygorvirus*, and *Gemykolovirus* were also assembled based on the reads of feces sample. However, no complete genome sequences of *Genomoviridae sp*. could be identified in the tissues. It is therefore possible that the *Genomoviridae* viruses were derived from the diet and might cause a local infection.

Parvoviruses are icosahedral, non-enveloped viruses with ssDNA genomes of about 5 kb in size, with prevalence in deep sequencing results of livestock showing diarrhea [26, 27]. The identified parvovirus with complete genome sequence in the present study belongs to the species *Galliform chaphamaparvovirus 1* of genus *Chaphamaparvovirus* of subfamily *Hamaparvovirinae* [28]. *Galliform chaphamaparvovirus 1* includes a single virus, turkey parvovirus 2, which was detected in the feces of domestic turkeys with high prevalence [29]. The present study expended the host spectrum of *Galliform chaphamaparvovirus 1*, and also supported that the host of the species limited to birds. Increasing evidences have shown that *Chaphamaparvoviruses* were localized in the gastrointestinal system and could play a potential role as an enteric pathogen associated with diarrhea [26, 27]. In addition, six partial sequences belonging to subfamily *Densovirinae* associated wild and zoo birds, and one partial sequence belonging to subfamily *Parvovirinae* associated bat were also identified in feces. *Parvovirinae* and *Densovirinae* were classic subfamilies of *Parvoviridae* defined in 1993 that infect vertebrate and invertebrate animals, respectively. The results showed that the wild birds in Dongying Biosphere Reserve carry a large number of parvovirus that infecting both birds and mammals, which may promote to the death of the young red-crowned crane. Given the migratory nature of wild birds and wide host range the parvovirus, undoubtedly that the viruses excreted in the feces also play a vital role in virus transmission in the ecological environment.

Of particular note is that the dead red-crowned crane with diarrhea symptoms was collected in Dongying Biosphere Reserve, Shandong Province, which is located at the Chinese eastern migration area for bird migration, containing the East Asia-Australasia migration route and the middle of the Western Pacific migration route. Birds migrate from south to north in the spring. Red-crowned crane is one of the representatives of migratory bird species in the area [30]. Therefore, the viruses in the feces excreted into the environment probably have important ecological impacts. The virome of tissues and feces of the dead captive red-crowned crane with diarrhea symptoms therefore provides clues for comparison to those of other birds or following diarrheal disease outbreaks nearby or in other migratory areas.

## Conclusion

Taken together, although the etiology for diarrhea of the dead red-crowned crane remains to be clarified, we have identified virus species from families *Picornaviridae* and *Parvoviridae* associated with enteric diseases in the tissues of the red-crowned crane in Dongying Biosphere Reserve, Shandong Province, China and feces of individual birds breeding at the corresponding captive breeding center. In particular, the complete genome sequence of picornavirus, genomovirus, and parvovirus further suggested that infectious viruses of these families were possibly presented in the diseased red-crowned crane. Our results enriched our understanding of the virome of birds and provide a baseline for elucidating the causative agent for diarrhea of the birds or following virological disease outbreaks.

## Data Availability

The datasets generated and/or analysed during the current study are available in Sequence Read Archive (SRA) database with the SRA accession No. SRR26126367 and No. SRR26057895 for feces and tissues respectively.

## Abbreviations

mNGS: Metagenomic next-generation sequencing
SIA: Sequence independent Amplification
Cp: Capsid protein
Rep: Replication-associated protein
